# BMI as an Independent Determinant of Spinal Alignment in Degenerative Scoliosis

**DOI:** 10.3390/diagnostics16162499

**Published:** 2026-08-07

**Authors:** Celine Carmen Akta, Lukas Schönnagel, Janina Serve, Athina Danovasili, Tom Folkerts, Isa Wendenburg, Bernhard Hoehl, Matthias Pumberger, Michael Putzier, Thilo Khakzad

**Affiliations:** Center for Musculoskeletal Surgery, Charité—Universitätsmedizin Berlin, Corporate Member of Freie Universität Berlin, Humboldt-Universität zu Berlin and Berlin Institute of Health, 10178 Berlin, Germany; lukas.schoennagel@charite.de (L.S.); janina-shanela.serve@charite.de (J.S.); athina.danovasili@charite.de (A.D.); tom.folkerts@charite.de (T.F.); isa.wendenburg@charite.de (I.W.); bernhard.hoehl@charite.de (B.H.); matthias.pumberger@charite.de (M.P.); michael.putzier@charite.de (M.P.); thilo.khakzad@charite.de (T.K.)

**Keywords:** spinal alignment, BMI, SVA, sex differences, degenerative scoliosis, sagittal spinal alignment, spinal deformity

## Abstract

**Background/Objectives**: Sagittal spinal alignment is a key determinant of function and quality of life in degenerative spinal disorders. While age is an established determinant of sagittal balance, the independent contribution of BMI remains insufficiently characterized, particularly in degenerative scoliosis cases. This study aimed to investigate the association between BMI and sagittal alignment, quantify its effect relative to age, and explore potential sex-specific differences. **Methods**: This retrospective single-center study included 293 adults with degenerative scoliosis who underwent standing full-spine EOS imaging. Multivariable linear regression models assessed the association between BMI and sagittal vertical axis (SVA), adjusting for age, sex, diabetes mellitus, and osteoporosis. Secondary analyses examined associations with coronal deformity (Cobb angle) and lumbar degeneration (Wilke score). Sex-stratified analyses and Receiver Operating Characteristic (ROC) analyses for sagittal decompensation were also performed. **Results**: The cohort had a median BMI of 26.2 kg/m^2^ (56% female; median age 71 years). Median SVA measured 56.4 mm (interquartile range 30.6–87.1), and 58% of patients exhibited sagittal decompensation. In sex-stratified analyses, higher BMI was independently associated with greater SVA in women (*p* = 0.015), whereas no significant association was observed in men (*p* = 0.061). ROC analysis demonstrated limited overall discrimination; however, a BMI ≥ 35 kg/m^2^ showed high specificity and positive predictive values exceeding 80% for sagittal decompensation. **Conclusions**: BMI is independently associated with sagittal alignment in degenerative scoliosis and contributes to sagittal decompensation to a degree comparable to age. These findings suggest that body weight represents a clinically relevant biomechanical factor that should be considered alongside age and spinopelvic parameters when interpreting sagittal alignment, although BMI’s limited discriminatory performance for sagittal decompensation precludes its use as a standalone screening tool.

## 1. Introduction

Sagittal spinal alignment is a key determinant of function, disability, and quality of life in patients with degenerative spinal disorders. In particular, sagittal decompensation has been linked with pain, reduced physical performance and health-related quality of life [1,2]. Restoration of sagittal alignment has been shown to improve postoperative function, pain, and patient satisfaction in degenerative adult spinal deformity [1,3], making the identification of modifiable determinants of sagittal balance a clinically relevant objective.

From a biomechanical perspective, an association between body mass index (BMI) and sagittal decompensation appears intuitive. Increased body mass alters axial loading, shifts the center of gravity anteriorly, and increases the muscular and structural demands required to maintain upright posture [4,5]. Additionally, an increased BMI also positively influences global muscle atrophy and paraspinal muscle volume, which further exacerbates sagittal decompensation [6,7]. However, despite this apparent plausibility, the relationship between BMI and sagittal alignment has not been adequately quantified, especially in patients with degenerative scoliosis. This population exhibits complex spinal geometry, segmental degeneration, and compensatory mechanisms that differ substantially from those of non-scoliotic or asymptomatic cohorts, limiting the generalizability of existing evidence.

Previous studies addressing sagittal alignment have predominantly focused on aging as the primary determinant of sagittal balance, often treating BMI as a secondary covariate or confounder rather than as an independent factor of interest [8,9]. As a result, the relative magnitude of the association between BMI and sagittal vertical axis (SVA) compared with established determinants such as age remains unclear. Furthermore, potential sex-specific differences in this association remain unexplored.

Clarifying the role of BMI in sagittal alignment is clinically relevant for several reasons. First, BMI represents a modifiable factor within conservative treatment strategies; weight reduction may influence sagittal balance and associated symptoms, especially in the context of increasing availability of effective pharmacological weight management strategies [10]. Second, contemporary alignment concepts and correction targets largely rely on age-adjusted parameters and do not account for the increased mechanical load and reduced muscle traction associated with a higher BMI. In these patients, uncritical application of such targets may result in overcorrection and further increase mechanical stress on the spinal construct.

Against this background, the present study aimed to investigate the association between BMI and sagittal alignment in patients with degenerative scoliosis. Specifically, we sought to quantify the effect of BMI on SVA, compare its magnitude to that of age, and explore potential sex-specific differences. By contextualizing BMI alongside established determinants of sagittal balance, this study seeks to inform both conservative management strategies and alignment considerations in this patient population.

## 2. Materials and Methods

### 2.1. Study Design and Population

This retrospective single-center study included patients who presented to our clinic between 2013 and 2023. Eligible participants were adults diagnosed with degenerative scoliosis (Cobb angle > 10°) on standing imaging. Patients were included if complete clinical data and full-length standing spinal imaging were available. Exclusion criteria comprised prior spinal deformity surgery, non-degenerative scoliosis, neuromuscular or congenital spinal disorders, and incomplete imaging or clinical records. The study was conducted in accordance with institutional ethical standards and approved by the local ethics board (EA2/310/23).

### 2.2. Imaging and Radiographic Assessment

As part of routine clinical care, all patients underwent standing full-spine imaging using EOS technology [11]; radiographic parameters were subsequently extracted retrospectively from these existing images rather than acquired specifically for this study. SVA was measured as the horizontal offset between the C7 plumb line and the posterior superior corner of S1. Sagittal decompensation was defined as an SVA greater than 50 mm. Coronal deformity was assessed using the Cobb angle [12]. Lumbar disc degeneration was evaluated at each lumbar level using the Wilke grading system and subsequently combined into a global degeneration score to reflect overall lumbar degenerative burden [13,14].

### 2.3. Clinical Variables

BMI, as documented in the medical record at the time of the clinically indicated imaging, was extracted retrospectively and treated as a continuous variable. For descriptive and Receiver Operating Characteristic (ROC) analyses, BMI was additionally categorized according to standard clinical thresholds. Age and sex were recorded for all patients. Comorbidities including diabetes mellitus and osteoporosis were extracted from patient medical records and included as covariates, as both are established contributors to bone and musculoskeletal health. Other potentially relevant confounders, including smoking status, physical activity level, and prior spinal interventions, were not consistently available in this retrospective cohort and could therefore not be included as covariates.

### 2.4. Statistical Analysis

Continuous variables are reported as median (interquartile range (IQR)), and categorical variables are reported as counts and percentages. Multivariable linear regression models were used to assess the association between BMI and continuous SVA, adjusting for age, sex, diabetes mellitus, and osteoporosis. To allow a comparison of effect sizes, continuous predictors were z-score normalized prior to modeling. Separate multivariable models were constructed to examine the association of BMI with coronal deformity (Cobb angle) and global lumbar degeneration (Wilke score). Sex-specific analyses were performed using stratified regression models. ROC analyses were conducted to evaluate the ability of BMI to discriminate sagittal decompensation (SVA > 50 mm). Positive predictive values were calculated for standard BMI categories, stratified by sex. Statistical significance was defined as *p* < 0.05. All analyses were performed using R Studio (Version 3.5.9).

## 3. Results

### 3.1. Demographic Characteristics

The cohort comprised 293 patients with degenerative scoliosis (129 men, 44%; 164 women, 56%); full demographic and radiographic characteristics are presented in Table 1. Age, BMI, and diabetes prevalence did not differ by sex. Women had a higher prevalence of osteoporosis (*p* = 0.002) and showed higher sacral slope, higher pelvic incidence, and more negative lumbar lordosis than men (all *p* < 0.05), while SVA did not differ by sex (*p* = 0.28). The major curve Cobb angle was slightly higher in women (*p* = 0.045).

### 3.2. Multivariable Regression Results

In multivariable linear regression analysis, BMI showed an independent association with SVA. After z-score normalization, the standardized effect of BMI on SVA was comparable in magnitude to that of age (*p* < 0.001 for both). Other covariates, including sex, diabetes mellitus, and osteoporosis, were not significantly associated with sagittal vertical axis. Variance inflation factors were consistently low, indicating no relevant multicollinearity.

For coronal deformity, BMI demonstrated a non-significant trend with the Cobb angle (*p* = 0.052). Notably, the magnitude of the BMI effect exceeded that of age (*p* = 0.086), despite limited overall model fit. Diabetes mellitus showed an independent negative association with the Cobb angle, while sex was positively associated (*p* > 0.05).

In analyses of lumbar degeneration, BMI was additionally significantly associated with the global Wilke degeneration score (*p* = 0.035), independent of covariates. However, age remained the dominant determinant of degeneration (*p* < 0.001). Full models are shown in Table 2.

### 3.3. Subgroup Analysis by Sex

In sex-stratified analyses, the association between BMI and SVA differed between men and women. In women, BMI remained significantly associated with increased SVA (*p* = 0.015), independent of age and comorbidities. In men, a similar effect size was observed, but the association did not reach statistical significance (*p* > 0.05). For coronal deformity, no significant association between BMI and Cobb angle was observed in either sex, although the direction of the effect was consistent with the main analysis. In analyses of lumbar degeneration, BMI was significantly associated with the Wilke degeneration score in women (*p* = 0.007), whereas no such association was observed in men (*p* = 0.898), which may reflect limited statistical power in this subgroup rather than the absence of a true association (Table 3).

To formally test whether the association between BMI and each outcome differed by sex, we additionally modeled a BMI × sex interaction term for each parameter. This interaction did not reach statistical significance for SVA (*p* = 0.929), Cobb angle (*p* = 0.598), or the Wilke degeneration score (*p* = 0.059), although the latter approached significance (Table 2). A similar borderline pattern was observed for lumbar lordosis (*p* = 0.084) and PI–LL mismatch (*p* = 0.087), suggesting a consistent, non-significant trend toward sex-specific effects across several sagittal parameters (Table 4). For sacral slope, the interaction term was statistically significant (*p* = 0.030), despite neither sex-stratified estimate reaching significance individually, indicating a true divergence in the direction of the BMI–SS association between men and women (Table 4).

### 3.4. ROC Analysis

**Figure 1:**
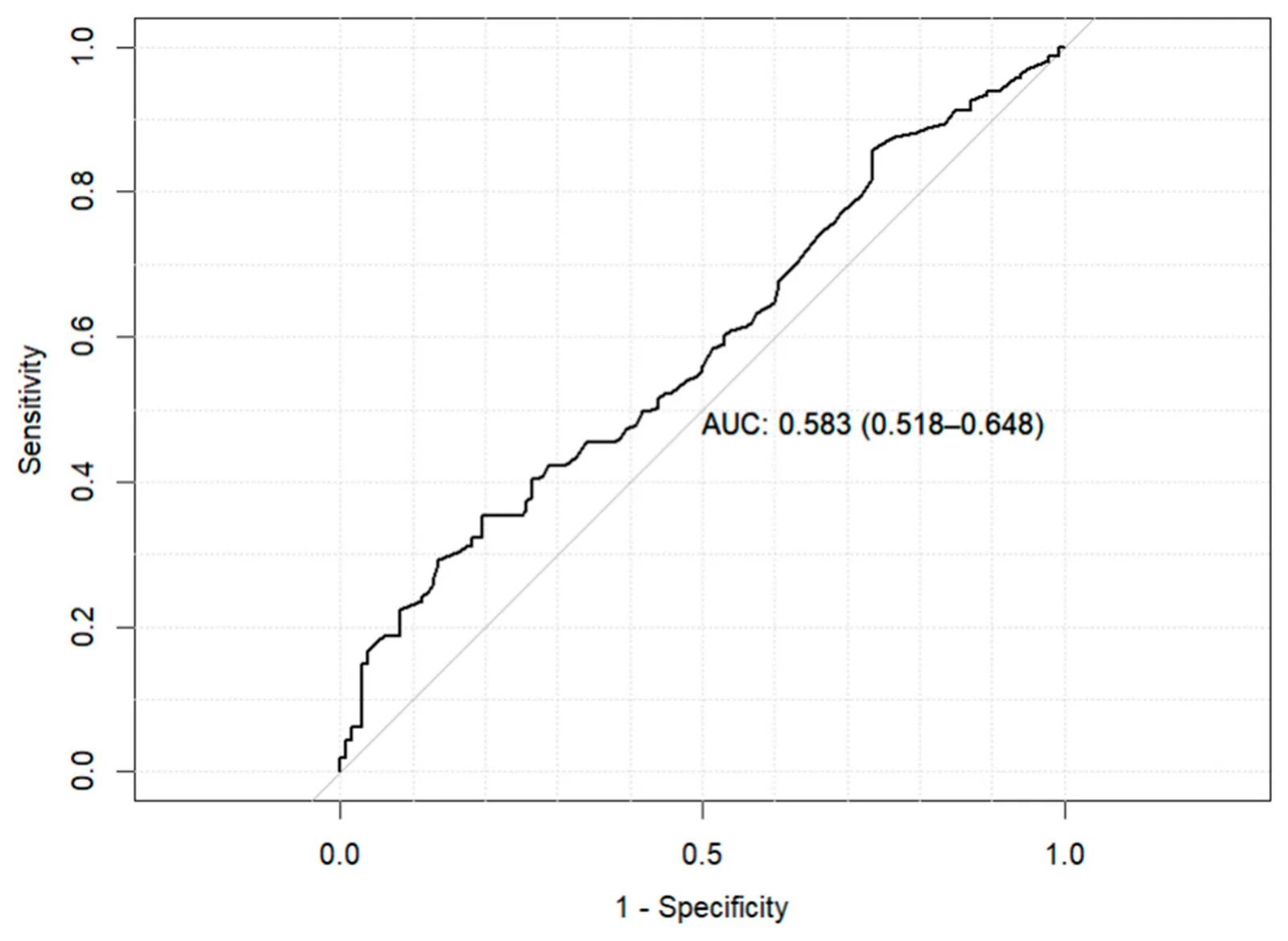
ROC analysis for BMI and sagittal decompensation: while body mass index (BMI) demonstrated low overall discriminative ability for sagittal decompensation (area under the curve (AUC) 0.583, 95% CI 0.518–0.648), increasing BMI thresholds were associated with a marked rise in specificity. Specificity increased from 0.02 at a BMI threshold of 20 kg/m^2^ to 0.82 at 30 kg/m^2^ and 0.97 at 35 kg/m^2^, with corresponding increases in positive predictive value (0.55, 0.68, and 0.80, respectively). This indicates BMI is not suitable for differentiation or screening, but may serve as a highly specific indicator at higher thresholds.

ROC analysis, using sagittal decompensation (SVA > 50 mm) as the binary outcome, showed limited overall discriminative performance of BMI (area under the curve (AUC) = 0.578), indicating that BMI alone cannot reliably distinguish patients from those without sagittal decompensation. Higher BMI thresholds were associated with high specificity and positive predictive values, at the cost of reduced sensitivity (Figure 1).

**Figure 2:**
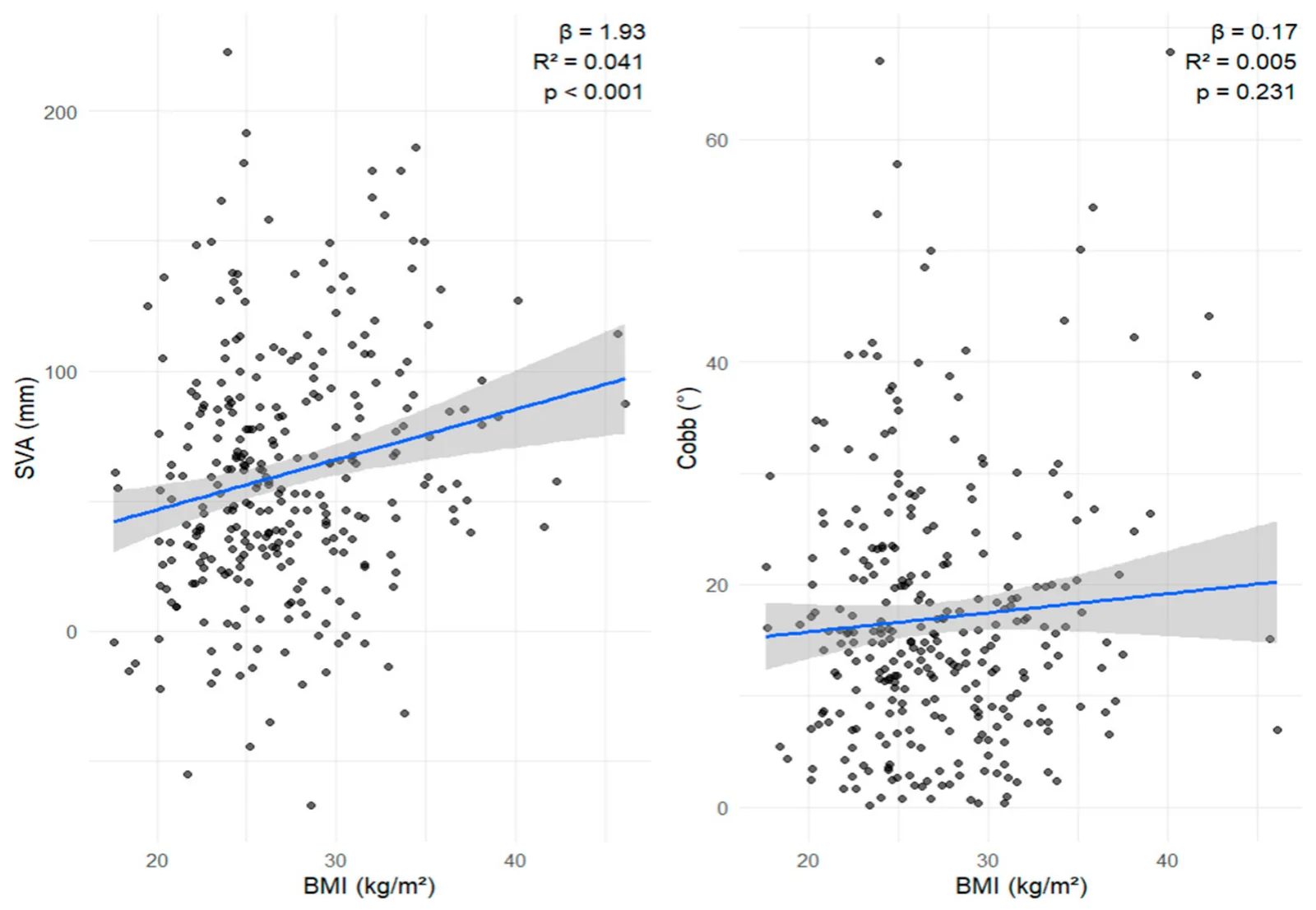
Association between BMI, SVA and Cobb angle: scatterplots illustrating the relationship between body mass index (BMI) and sagittal vertical axis (SVA; left panel) as well as coronal deformity measured by Cobb angle (right panel). Blue lines represent fitted linear regression models with 95% confidence intervals (shaded areas). BMI showed a stronger association with sagittal alignment (SVA) than with coronal deformity.

In the overall cohort, a BMI threshold of 35 kg/m^2^ was associated with a positive predictive value of 0.80 and a specificity of 0.97, while sensitivity was low. Similar patterns were observed in sex-stratified analyses. In women, a BMI threshold of 35 kg/m^2^ yielded a positive predictive value of 0.82 with high specificity (0.96), whereas in men the same threshold was associated with high specificity (0.98) and a lower positive predictive value (0.67). Lower BMI thresholds increased sensitivity at the expense of specificity (Figure 2).

## 4. Discussion

This study demonstrates an independent association between BMI and sagittal decompensation in patients with degenerative lumbar scoliosis. The magnitude of the association between BMI and SVA was comparable to that of age, indicating that body weight represents a clinically relevant factor in sagittal alignment. This finding highlights that BMI is not merely a background characteristic, but a meaningful factor associated with sagittal balance in this patient population, especially in women. This relationship accounts for only a modest proportion of the overall variability in sagittal alignment. Although the association between BMI and Cobb angle did not reach statistical significance, effect size exceeded that of age; therefore, this finding should be regarded as exploratory rather than conclusive evidence. Increased age was more strongly associated with lumbar degeneration than BMI; this was expected given the established role of age as a primary determinant of degenerative changes. However, BMI showed one third of the effect size for age, suggesting a potentially clinically relevant contribution to lumbar degeneration. Women showed a stronger association with BMI and SVA, which may reflect sex-specific differences in muscle mass, muscle composition, and load distribution. Beyond SVA, women showed independent associations between BMI and both lumbar lordosis and PI–LL mismatch, whereas no such associations were observed in men, though the corresponding interaction terms did not reach significance. For sacral slope, the interaction analysis was significant despite null individual estimates, suggesting a true sex-specific divergence. A plausible common explanation lies in sex differences in paraspinal musculature: compared with men, women have significantly smaller paraspinal muscles, and their sagittal alignment appears more dependent on muscle integrity, with paraspinal cross-sectional area inversely correlated with SVA and fatty infiltration positively correlated with SVA [15,16]. Menopause-related estrogen deficiency further compounds this vulnerability by accelerating disc degeneration, bone loss, and sarcopenia concurrently, creating a biomechanical environment in which increasing BMI has a disproportionate effect on sagittal balance [16,17].

Lower absolute muscle strength and differences in paraspinal muscle characteristics may reduce compensatory capacity under increased mechanical load, particularly in the presence of degenerative scoliosis. However, these mechanisms cannot be directly evaluated within the present analysis, as BMI does not distinguish fat mass from muscle mass and does not capture paraspinal muscle quality. A growing body of studies in the literature has linked sagittal imbalance to spine-specific sarcopenia: particularly reduced cross-sectional area and fatty infiltration of the multifidus and erector spinae [18]. Because BMI reflects body mass irrespective of its composition, part of the association observed between BMI and SVA, and its apparent sex-specific magnitude, may therefore be confounded with differences in paraspinal muscle quality rather than adiposity alone.

These findings suggest that BMI is an independent contributor in sagittal alignment, coronal deformity, and degenerative burden in patients with degenerative lumbar scoliosis. Most prior work on sagittal alignment in degenerative conditions and adult spinal deformity has focused on spinopelvic geometry, compensatory mechanisms, and age-related alignment changes, with BMI commonly treated as a covariate rather than as a primary exposure of interest [19]. The present study specifically quantifies BMI as an independent correlate of sagittal alignment, thereby addressing an underexplored aspect of degenerative scoliosis.

In adult spinal deformity cohorts, BMI has more frequently been studied in relation to surgical alignment targets and postoperative radiographic or health-related quality-of-life outcomes than as a determinant of pre-treatment sagittal malalignment. Focusing on treatment-related outcomes, Horn et al. investigated the effect of BMI on achieving age-adjusted alignment targets and their clinical benefit, rather than on baseline SVA [4]. Agarwal et al. used the same approach and assessed BMI in relation to postoperative changes in SVA and patient-reported outcomes after deformity surgery [20]. Both of these studies report inferior radiographic and clinical outcomes in patients with higher BMI. However, these studies do not address whether standard alignment concepts adequately reflect the biomechanical demands associated with increased body weight.

Beyond deformity surgery, population-based and mixed symptomatic cohorts have reported associations between obesity and radiographic alignment parameters. Takeuchi et al. demonstrated that higher BMI in community-dwelling older adults is associated with worse sagittal balance and more advanced degenerative spinal changes [5]. Biomechanical studies further support these findings by showing that increasing BMI leads to greater lumbar intervertebral disc deformation under dynamic loading conditions [5,21]. Together, these data support a mechanical link between body weight and sagittal alignment. Against this background, the present findings add to the literature by quantifying BMI as an independent correlate of SVA in a degenerative scoliosis cohort.

It is well established that increased body mass alters axial loading and bending moments acting on the spine and increases the demands placed on postural control to maintain sagittal balance [5,22]. In patients with degenerative scoliosis, compensatory capacity is often already limited by structural deformity, segmental degeneration, and reduced spinal flexibility in the affected segments [23]. Under these conditions, additional load and a higher BMI may have a disproportionate effect on sagittal alignment compared with non-deformed spines. This helps explain why sagittal decompensation was more strongly associated with BMI than coronal deformity, as coronal plane changes are likely driven predominantly by local degeneration, asymmetry, and segmental instability rather than by global loading alone.

Obesity is increasingly being recognized as a chronic condition rather than a readily modifiable perioperative factor [24,25]. Its chronicity is driven by genetic, biological, environmental, and behavioral factors, resulting in persistent metabolic dysfunction and systemic inflammation [24,25]. Evidence from the arthroplasty literature indicates that patients with obesity often do not achieve meaningful or sustained weight reduction following major joint replacement, despite functional improvement [26,27]. This suggests that BMI should be regarded as a relatively stable biomechanical condition rather than a dynamic parameter expected to improve after surgery. In this context, the consistently reported association between high BMI and inferior outcomes after adult spinal deformity surgery may partially reflect a mismatch between standard age-adjusted alignment targets and the persistent mechanical demands imposed by higher body weight.

Current alignment concepts are largely based on age-adjusted normative targets and do not explicitly account for differences in axial loading associated with obesity [9,28,29]. Applying these targets without consideration of BMI may result in alignment goals that are biomechanically insufficient in patients with high body weight, potentially contributing to persistent sagittal imbalance or mechanical complications. While the present study cannot establish causality or define BMI-adjusted alignment targets, the observed associations support consideration of body weight as an integral component of the biomechanical framework in which sagittal alignment is interpreted.

Given that sagittal decompensation is a multifactorial phenomenon influenced by degeneration, muscle capacity, and compensatory mechanisms, and the overall discriminative performance of BMI was limited, indicating that BMI alone cannot reliably distinguish patients with and without sagittal decompensation. Nevertheless, higher BMI thresholds were associated with high specificity and positive predictive values. In particular, more than 80% of patients with a BMI greater than 35 exhibited sagittal imbalances. Therefore, BMI should not be used as a screening parameter, but elevated BMI may nonetheless prompt closer radiographic evaluation in individual patients.

### Strengths and Limitations

This study benefits from a well-characterized cohort of patients with degenerative lumbar scoliosis evaluated over a long inclusion period, using standardized standing full-spine EOS imaging. The availability of comprehensive radiographic parameters allowed a detailed assessment of sagittal and coronal alignment as well as lumbar degeneration. Multivariable analyses with z-score-normalized predictors enabled direct comparison of the relative contributions of BMI and age, which strengthens the interpretability of effect magnitudes. In addition, sex-stratified analyses provided insight into potential sex-specific patterns that are often underexplored in deformity research.

A few limitations should be acknowledged. The retrospective design precludes causal inference, and residual confounding by unmeasured factors such as muscle quality, physical activity, T1 pelvic angle, global tilt or pain-related posture cannot be excluded. Functional outcomes and patient-reported measures were not available in this retrospective cohort; as a result, it remains unclear whether the observed radiographic differences translate into meaningful differences in pain, disability, or quality of life. Other potentially relevant confounders, such as smoking status, physical activity level, and prior spinal interventions, were similarly unavailable and could not be adjusted for, which may account for residual confounding. Furthermore, sagittal alignment was assessed at a single time point, preventing evaluation of temporal relationships or progression. As a single-center study, generalizability to other populations and care settings may be limited. BMI does not distinguish fat mass from muscle mass or capture paraspinal muscle quality or fat distribution; direct measures of muscle cross-sectional area or fatty infiltration were not available in this cohort, and therefore sex-specific differences in body composition could not be directly evaluated and may partly explain the observed sex-specific associations between BMI and SVA.

## 5. Conclusions

BMI shows a consistent and independent association with sagittal alignment in degenerative lumbar scoliosis, with an effect magnitude comparable to that of age for sagittal decompensation. These findings indicate that body weight, alongside age and spinopelvic parameters, should be considered when interpreting sagittal alignment in this patient population; however, considering the study design, they cannot inform surgical alignment targets at this stage. These findings support the need for prospective, longitudinal studies to determine whether alignment concepts that account for BMI can improve long-term balance and outcomes, before such approaches are incorporated into alignment planning or treatment algorithms in this patient population.

## Figures and Tables

**Table 1:** Demographic characteristics.

Variable	All (*n* = 293)	BMI < 30 (*n* = 217)	BMI ≥ 30 (*n* = 76)	*p* Value
Age [years]	71 (61–78)	71 (61–78)	70 (61–79)	0.935
Male	129 (44.0%)	101 (46.5%)	28 (36.8%)	0.183
Female	164 (56.0%)	116 (53.5%)	48 (63.2%)	
BMI [kg/m^2^]	26.2 (23.9–30.1)	24.8 (23–26.9)	33.15 (31.1–35.1)	**<0.001**
Diabetes Mellitus	57 (19.5%)	29 (13.4%)	28 (36.8%)	**<0.001**
Osteoporosis	21 (7.2%)	14 (6.5%)	7 (9.2%)	0.586
SS [°]	33.7 (27.1–40.3)	33.4 (27.2–40.7)	34.05 (26.3–39.68)	0.991
PI [°]	52.3 (45.4–62.5)	51 (44.1–62.5)	54.5 (47.62–62.42)	0.128
LL [°]	−44.7 (−54.7–−32.7)	−44.7 (−54.7–−33.6)	−44.65 (−54.52–−30.52)	0.656
SVA [mm]	56.4 (30.6–87.1)	50.8 (27.5–82.7)	74.65 (43.5–106.55)	**0.001**
Cobb [°]	14.9 (8.6–22.2)	14.9 (8.6–22.8)	14.95 (8.4–19.85)	0.779

Demographic, clinical, and radiographic characteristics of the study cohort stratified by body mass index (BMI < 30 vs. BMI ≥ 30). Except for sagittal vertical axis and diabetes mellitus, no significant differences were observed between BMI groups (*p* < 0.05). Predictor variables were z-score standardized prior to analysis; outcome variables were not standardized. Between-group comparisons were performed using the Mann–Whitney U test for continuous variables and the χ^2^ test for categorical variables. *p*-values below 0.05 were defined as significant. BMI = body mass index; SS = sacral slope; PI = pelvic incidence; LL = lumbar lordosis; SVA = sagittal vertical axis; Cobb = Cobb angle. Bold for all statistically significant *p* values to highlight importance.

**Table 2:** Regression Analysis.

+	SVA		Cobb		Wilke Score	
	Estimate	η^2^	Estimate	η^2^	Estimate	η^2^
BMI	**9.11 (3.63–14.60), *p* = 0.001**	0.036	1.42 (−0.01–2.86), *p* = 0.052	0.013	**0.12 (0.01–0.24), *p* = 0.035**	0.015
Age	**10.73 (5.65–15.81), *p* < 0.001**	0.056	1.16 (−0.16–2.49), *p* = 0.086	0.010	**0.34 (0.23–0.45), *p* < 0.001**	0.114
Sex	−6.21 (−16.65–4.23), *p* = 0.245	0.005	**2.92 (0.20–5.65), *p* = 0.036**	0.015	0.14 (−0.08–0.36), *p* = 0.222	0.005
Diabetes Mellitus	9.57 (−4.12–23.26), *p* = 0.172	0.006	**−5.39 (−8.96–−1.82), *p* = 0.003**	0.030	−0.27 (−0.56–0.02), *p* = 0.073	0.011
Osteoporosis	9.84 (−10.35–30.04), *p* = 0.340	0.003	5.13 (−0.14–10.40), *p* = 0.057	0.013	0.25 (−0.18–0.68), *p* = 0.256	0.004
Model r^2^	0.102		0.056		0.120	

Multivariable regression analysis of factors associated with sagittal vertical axis (SVA), coronal deformity (Cobb angle) and Wilke score; sample size remains constant amongst tests. Body mass index (BMI) and age showed significant and comparable association with increased SVA and lumbar degeneration, whereas their influence on Cobb angle was smaller and did not reach statistical significance. In contrast, Cobb angle was more strongly associated with sex and diabetes mellitus, while SVA was less affected by these variables. Effect sizes were small-to-moderate, indicating a stronger overall influence of covariates on sagittal alignment than on coronal deformity. Bold for all statistically significant *p* values to highlight importance.

**Table 3:** Subgroup analyses.

	Females		Males	
SVA	Estimate	η^2^	Estimate	η^2^
BMI	**8.53 (1.75–15.30), *p* = 0.015**	0.037	9.06 (−0.35–18.47), *p* = 0.061	0.028
Age	**9.28 (1.78–16.77), *p* = 0.016**	0.036	**12.89 (5.73–20.04), *p* < 0.001**	0.091
DM	12.71 (−7.30–32.71), *p* = 0.215	0.01	6.48 (−12.16–25.12), *p* = 0.497	0.004
Osteoporosis	11.75 (−10.70–34.21), *p* = 0.306	0.007	−6.28 (−65.35–52.79), *p* = 0.835	0
Model r^2^	0.086		0.105	
**Cobb**				
BMI	1.58 (−0.32–3.49), *p* = 0.105	0.016	0.89 (−1.23–3.02), *p* = 0.411	0.005
Age	1.23 (−0.88–3.34), *p* = 0.256	0.008	1.20 (−0.41–2.82), *p* = 0.147	0.017
DM	**−5.10 (−10.73–0.53), *p* = 0.078**	0.019	**−5.66 (−9.87–−1.46), *p* = 0.009**	0.053
Osteoporosis	5.47 (−0.85–11.78), *p* = 0.092	0.018	1.96 (−11.37–15.30), *p* = 0.773	0.001
Model r^2^	0.021		0.032	
**Wilke Score**				
BMI	**0.17 (0.05–0.29), *p* = 0.007**	0.044	−0.02 (−0.25–0.22), *p* = 0.898	0
Age	**0.35 (0.22–0.49), *p* < 0.001**	0.141	**0.34 (0.16–0.52), *p* < 0.001**	0.1
DM	−0.10 (−0.46–0.26), *p* = 0.585	0.002	−0.44 (−0.90–0.03), *p* = 0.068	0.027
Osteoporosis	0.28 (−0.12–0.69), *p* = 0.173	0.012	−0.21 (−1.69–1.27), *p* = 0.781	0.001
Model r^2^	0.159		0.084	

Sex-stratified multivariable regression analyses for sagittal vertical axis (SVA), coronal deformity (Cobb angle) and Wilke score. Overall, associations between covariates and SVA demonstrated larger effect sizes in female participants, particularly for body mass index (BMI) and age, whereas effects in males were primarily driven by age. In contrast, associations with Cobb angle were generally weaker in both sexes, with diabetes mellitus (DM) showing a moderate effect in males only. For the Wilke degeneration score, BMI was independently associated with lumbar degeneration in women but not in men, whereas age remained the dominant determinant of degeneration in both sexes. Bold for all statistically significant *p* values to highlight importance.

**Table 4:** Regression Analysis: BMI and spinopelvic parameters.

Parameter	Overall Estimate	η^2^	Male Estimate	Female Estimate	BMI × Sex Interaction	Model R^2^
SS	−0.69 (−2.15–0.76), *p* = 0.351	0.003	1.46 (−0.83–3.75), *p* = 0.213	−1.56 (−3.47–0.35), *p* = 0.111	**−3.41 (−6.46–−0.35), *p* = 0.030**	0.020
PI	0.78 (−0.91–2.46), *p* = 0.367	0.003	0.53 (−2.33–3.39), *p* = 0.718	0.96 (−1.19–3.12), *p* = 0.383	−0.03 (−3.60–3.54), *p* = 0.986	0.056
LL	−1.91 (−3.94–0.11), *p* = 0.065	0.012	0.37 (−2.98–3.72), *p* = 0.829	**−2.86 (−5.47–−0.25), *p* = 0.033**	−3.76 (−8.03–0.50), *p* = 0.084	0.044
PI–LL Mismatch	**2.69 (0.67–4.72), *p* = 0.010**	0.023	0.17 (−2.99–3.33), *p* = 0.918	**3.82 (1.14–6.51), *p* = 0.006**	3.73 (−0.53–7.99), *p* = 0.087	0.100

Sex-stratified multivariable regression analyses for additional spinopelvic parameters (sacral slope (SS), pelvic incidence (PI), lumbar lordosis (LL), and PI–LL mismatch), adjusted for age, diabetes mellitus, and osteoporosis. As with SVA, BMI was independently associated with LL and PI–LL mismatch in women but not in men. For SS, the formal BMI × sex interaction term was statistically significant (*p* = 0.030), despite neither sex-stratified estimate reaching significance individually, indicating a true divergence in the direction of the BMI–SS association between men and women. T1 pelvic angle and global tilt were not measured in this cohort. Bold for all statistically significant *p* values to highlight importance.

## Data Availability

The original contributions presented in this study are included in the article. Further inquiries can be directed to the corresponding author.

## Abbreviations

The following abbreviations are used in this manuscript:

AUC	Area Under the Curve
BMI	Body Mass Index
CI	Confidence Interval
IQR	Interquartile Range
LL	Lumbar Lordosis
PI	Pelvic Incidence
ROC	Receiver Operating Characteristic
SS	Sacral Slope
SVA	Sagittal Vertical Axis
